# Amphibious ethics and speculative immersions: laboratory aquariums as a site for developing a more inclusive animal geography

**DOI:** 10.1080/14702541.2024.2378308

**Published:** 2024-07-16

**Authors:** Beth Greenhough, Emma Roe, Reuben Message

**Affiliations:** a School of Geography and the Environment, University of Oxford, Oxford, UK; b School of Geography and Environmental Science, University of Southampton, Highfield, UK

**Keywords:** Animal geographies, marginalisation, fish, empathy, ethics

## Abstract

Human capacity to sense and respond to the suffering of non-human animals is key to animal care and welfare. Intuitively these modes of relating seem best suited to interactions between humans and warm-blooded mammals who share human-like facial features and characteristics. Animal geographers and those working in animal welfare have noted the challenges that humans face in learning to care about fishes, and how this leads to welfare guidelines and regulations which are poorly suited to aquatic species. This paper draws on interviews with laboratory aquarists and biomedical researchers to explore how they have learnt to sense and respond to the needs of fishes in the laboratory. We offer two key observations. Firstly, despite significant bodily differences, humans find ways to empathise with fishes. Secondly, whilst observations of bodies and behaviours predominate in laboratory mammal welfare assessments, when working with fishes water quality serves as an important proxy for species health. We conclude that the laboratory aquarium signifies methodological and conceptual limits in contemporary animal geographies. We further argue that these barriers should be understood as cultural, and – as we demonstrate – that there is consequently scope and capacity to reach beyond them by engaging in amphibious ethics and speculative immersions.

## 1. Introduction

In the 30 years since Philo (1995) made the claim that animals were marginalised in human geography, there have been considerable efforts to bring the animals back in. However, within this body of work, fishes and many other aquatic species still rarely feature (for exceptions see Bear & Eden, 2011; Bull, 2011; Johnson, 2015; Jones, 2000; Liao, 2024; Waley, 2000). Fishes appear to slip to the margins of human emotional, moral and ethical imaginations; under the water, out of sight, out of mind. Fishes are inhabitants of watery spaces – oceans, rivers, seas and lakes – that have been historically cast as empty and meaningless (Price, 2004), allowing ‘them to be colonised without acknowledgement of prior claims to those landscapes’ (Trudeau & McMorran, 2011, p. 444).^1^ Consequently, scholarship on aquatic species in and beyond animal geography has focused on the challenges people seem to have (at least in many Western cultures^2^) when it comes to relating empathetically with fishes. Jones (2000, p. 284), for example, suggests that ‘our (human) ethical imaginations also find water a hostile, impenetrable space, and that as a result many of the lives lived there are ethically invisible to us’, while Driessen (2013, p. 253) observes that empathy for fishes just appears ‘beyond human capacity’. In the face of changes in the intensity of human-fish interactions globally – including the over-exploitation of fish stocks, the growth of fish and octopus farming, the exotic pet trade and the rise of fish as mainstay laboratory models – a growing number of commentators have identified this challenge as a problem and, in response, explored both ways of reimagining popular understandings of fishes and the potential for new kinds of human-fish relations and ethics (see, for example, Balcombe, 2017; Lien, 2015; Message, 2024; Probyn, 2015, 2016; Wadiwel, 2016).

These concerns connect with Ginn et al.’s (2014) call for attention to how we, humans, might flourish with ‘awkward creatures’. Like the silent majority of invertebrates often neglected in animal studies and cognate research (Moore & Wilkie, 2019), fish are ‘critters’ who are often defined (and subsequently marginalised) due to their non-mammalian bodies and features. Futhermore, fish are set further apart by the watery environs that they inhabit, which means that to consider fish means thinking with both bodies and their contexts. The recent ‘blue turn’ in the environmental humanities and social sciences posits the aquatic as a new medium which brings into question not only the bodies we – as humans (albeit humans of many different kinds) – think with and through, but also the implicit ‘landedness’ of our ways of thinking in terrestrial landscapes and airy atmospheres (Helmreich, 2015; Jue, 2020; Peters, 2020; Steinberg & Peters, 2015). This questioning makes fishes an interesting case through which to engage with what inclusivity might mean for animal geographies: what practices and processes emerge in order to care for species whose bodies, behaviours and habitats have historically been cultured as alien and less meaningful to our own? As the Anthropocene radically transforms watery worlds, there is, we suggest, an increasingly urgent need to prioritise embodied, aquatic, somatic sensibilities (Greenhough & Roe, 2011) or what we term (drawing on Chao, 2020) an amphibious ethics generated through practices of bodily attunement in and through the medium of water.

The capacity to learn to be affected by other species, usually at moments of co-presence, is a skill developed by those responsible for animal care and welfare. Over a decade ago, Greenhough and Roe (2011) argued for the role of somatic sensibilities – empathy derived from a shared experience of having a body – in understanding how humans, and specifically laboratory animal technologists, can relate to the care needs of a nonhuman other. In this paper, we consider the value of this concept for understanding how humans become sensitised to the care and welfare needs of aquatic animals, specifically fishes.^3^ We take as our empirical case the practice of caring for fishes, predominantly zebrafishes, in animal research aquariums. Animals living under water are less easy to observe and have body shapes, sizes and capacities markedly different to humans. Yet despite this challenge, our research in academic research aquariums found that interspecies bodily ‘attunement’ (Despret, 2004) does occur in aquatic spaces, albeit in complex ways, and often involves translating skills developed through interacting with terrestrial, mammalian (mostly murine^4^) bodies, often with ambiguous results (see also Message, 2024).

The humans who we observed caring for fishes combined an ability to become ‘attuned’ to variations in the visual aesthetics of fishes’ bodies and behaviours with an equally careful attention to the contained environment that the fishes lived within. The latter is striking. The quality of a terrestrial animal’s habitable world – housing, substrate, food, air temperature (if not air quality), access to water – is an established concern in animal welfare, but daily animal health checks often resort to an animal body and behaviour assessment. While mandatory daily health and welfare checks focused on bodies and behaviours are also conducted for all aquatic animals in laboratories, at the same time staff are engaged in a continuous programme of monitoring and adjusting environmental parameters, considered equally if not more important to understanding colony status. The relative porosity of the boundary between care for the bodies of fishes and care for water quality suggests the possibility of bringing together the concerns of animal (fish) welfare with a broader concern for the aquatic environments within which they thrive. Notably, this can seem ever-present in laboratory practices for forms of living cellular life where there is care for chemical media (Landecker, 2016) as their life-giving environment.

We begin with an overview of why we think relations between humans and fishes, especially in the context of laboratory animals, should still be considered a socially marginalised form of human-animal relation (in contrast to pets, farm and wild mammals). Whether under the surface of water or deliberately hidden within bioscience buildings, the marginality of these relations has repercussions for how human geographers have (or have not) accounted for their lives, offering us scope to ask questions about species inclusivity. We then contextualise our empirical case, charting the rise of fishes, and zebrafishes in particular, as a laboratory model, as well as details of how we traced this emergence methodologically. Two more substantive sections then follow which explore respectively: (i) how those working with fishes in research laboratories learn to relate to them; and (ii) how within the laboratory aquarium caring for the animal other – fishes – is inseparable from caring for the wider aquatic environment within which they are immersed. This second section will show how environmental parameters serve as both proxy for and bridge towards a mode of relating grounded in bodied symphysis, a cross-species moral accountability derived from shared experiences of having a sensing, feeling body (Acampora, 2006). We contend that contemporary laboratory animal care practices for aquatic species, who sit on the spatial and cultural margins of species knowable and relate-able to humans, offer an empirical resource for studying both the interplay between (em)bodied and environmental care ethics and for thinking through how social concerns for inclusivity can be relevant to animal geographies.

## 2. Conceptualising marginalised relations between humans and fishes

Learning to be affected by animals, as a method for attending to their interests and care needs, has been a key theme in animal studies and the environmental humanities. Tsing (2015) writes of the ‘arts of noticing’, to bring our attention to the lifeworlds and liveliness of other-than-human beings; Rose (2011, p. 42) draws on a minority reading of Levinas to stress the importance of meeting face-to-face with animal others as a means of locating ‘ethical call-and-response within the living reality of the material world’; Despret (2004) writes of empathy as emerging through processes of mutual attunement, as human and non-human bodies both affect and learn to be affected by each other; and Greenhough and Roe (2011) draw on Acampora’s (2006) work on symphysis and corporeal compassion (as a form of empathy derived from the shared experience of having a body) to advocate for the recognition of somatic sensibilities as playing a key role in informing animal caretakers’ decisions about when something is wrong with an animal in their care. Driessen (2013, p. 252) suggests, however, that ‘[t]hese types of relational understandings tend not to favour fish’. Intuitively these various modes of relating seem best suited for human interactions with animals who are ‘big like us’, or who have particular cultural-spatial histories or bodily characteristics that make them charismatic and relatable (Hird, 2010; Lorimer, 2015).

In the work of animal geographies, animals feature as both givers and recipients of care (see, for example, Arathoon, 2023; Davies et al., 2018; Donald, 2019; Gorman, 2019; Nelson, 2017; Taylor & Carter, 2020) but with a predominantly mammalian – indeed, diurnal and land-dwelling – focus (albeit with some notable exceptions: see, for example, Bear, 2019; Ginn et al., 2014; Lorimer, 2015). Elsewhere we have explored the value of bringing work on institutional cultures of care for humans and for animals into conversation (Greenhough et al., 2023). Here we reflect more on how, *contra* much of the work looking at care for human (and some mammalian) subjects where the need for care is often seen as intuitive or implicit, the idea of fishes as subjects of and deserving of care is in and of itself a case that needs to be made. After all, as we have rhetorically argued elsewhere, ‘it’s just a fish’, isn’t it’ (Message & Greenhough, 2019)?

If we combine this focus on the challenges that humans face relating to fishes and the centrality of attunement to understanding how care is achieved in human-animal relations, two further readings of the literature emerge. Firstly, we find a growing hope for humans learning to attune to fishes and other ‘awkward creatures’ (Ginn et al., 2014). Ginn (2014), for example, is optimistic about the extent to which somatic sensibilities still holds merit in human-slug encounters, where gardeners have been known to acknowledge shared-but-different corporeal vulnerability across species lines. Hazard (2022, p. 19), moreover, argues that the alienation of humans from fishes and their worlds is manufactured not intrinsic, agreeing with Price (2004) that it is a product of settler-colonial-science practices of extraction and abstraction that ‘severed relations between people, fishes, and the waters they swim in’. Hazard therefore holds open the possibility of rebuilding relations. Scholars have also described situations where humans have learnt to love jellyfish (Johnson, 2015), to reach out to cup-corals with fingeryeyes (Hayward, 2010), and, most relevant here, to approach empathy for fishes (Allmark-Kent, 2021). This finding resonates with scholarship in geography that has explored: how human relations with genetically engineered zebrafish are shaped by the aesthetic (as well as scientific) values attached to florescent zebrafish, alert to the tentative but elusive possibilities opened up here for ethnographic exploration (Davies, 2014); the embodied and multisensory ways in which recreational anglers encounter fish and their watery environments (Bear & Eden, 2011); or the empathy that shapes hunter-prey encounters between Sami fishers and ‘fish in the river’ in Lapland (Krause, 2014, p. 263).

In contrast, others emphasise that, conditioned by culture and physiology, the experience of species who live an embodied existence radically other than our own can lead to incomprehension, withdrawal and detachment (compare Schrader, 2015). Indeed, it is likely only in specific circumstances that such detachment can be meaningfully overcome. Nevertheless, we question the analysis that leads Ginn et al.’s (2014, p. 16) insistence that the ‘irreducible strangeness’ present in all inter-species meetings represents a distinct challenge to more embodied approaches to care or indeed to an ethics of care reliant on face-to-face encounters (see also Cusworth, 2023; Davies, 2012). Our findings suggest otherwise, and by dwelling on their strangeness, these sentiments risk perpetuating the marginalisation of some species. Instead, we follow Helmreich (2009, p. 17), who, in his writings about ocean-dwelling microbes, reminds us that the figure of the alien does not signify an absolute other but rather ‘life forms whose place in our forms of life is yet to be determined’. The experience of laboratory lives offers one route through which this angle might be explored.

Secondly, responding to Jue’s (2020) call for a media-specific approach to the production of knowledge and representation, we examine possibilities for materially and experientially attuning our bodies to the aquatic environments within which fishes reside. Here we find inspiration in: Hazard’s (2022, p. 10) work on underflows which emphasises the entanglement of both land and water; Steinberg and Peters’s (2015) call for thinking though wet ontologies and fluid spaces as a challenge to terrestrial bias; Fredriksen’s (2019) reading of the inter-tidal zone as a site of more-than-human encounter; and Anand’s (2023) anthroposea, which documents places where humans have learnt to dwell amphibiously with nonhuman others. Neimanis (2012, pp. 86–87), meanwhile, reminds us that water may be a medium of connection as well as separation between human and other-than-human bodies and worlds: when we drink we ‘ingest the ghosts of bodies that haunt the water’, and when we urinate we return to the sea remnants of our consumption as traces of drugs and hormones; ‘[w]ater connects the human scale to all other scales of life, both unfathomable and imperceptible’. As Helmreich (2009, p. 5) also notes, water bodies – like the ocean – have a ‘fluid capacity to link the smallest microorganism to the largest ecosystem’. Through what these works suggest, water becomes materially heterogeneous and promiscuous. Of course, water may not literally connect humans and fishes in laboratory aquariums, but, as previous work on angling has shown, including Bear and Eden’s (2011) focus on angler’s ‘watercraft’ and Krause’s (2014, p. 362) focus on ‘fish in the water’, attuning to water becomes a means of beginning to attune to fishes.

Inspired by work on the importance of contact zones as sites for thinking through the political-ethical potentialities of human-animal encounters, we position the laboratory aquarium as a site where ‘practical, embodied knowledge’ (Fredriksen, 2019, p. 762) is developed which allows humans better to notice and attune to zebrafish and their environs. We acknowledge how the possibilities offered by such space are shaped and limited by the (often radically unequal) power relations at play (Wilson, 2019): the vast majority of research using zebrafish is instrumentally driven by seeking knowledge to improve human health. But, in contrast to work sceptical about the possibilities of encounters within contained and controlled spaces where animals are kept in captivity (Kirksey, 2015) or which associates lab spaces with objective, detached, abstract forms of knowledge production (Fredriksen, 2019), we see labs as sites with the potential for generating empathy and somatic sensibilities across difference (Greenhough & Roe, 2011; Haraway, 2008). Where Wilson (2019) might focus on how zebrafish are out-of-place and subordinated in a human-dominated research facility, we pay attention to how humans too are rendered physically and emotionally less than comfortable through the labour of maintaining zebrafish in an emergent laboratory ecology (Kirksey, 2015), a labour which in turn opens up possibilities for empathy.

Furthermore, thinking of water as a medium for contact – an opportunity for connection, rather than separation – enables seeing it as a space open to the development of ecologies of care analogous to those Puig de la Bellacasa (2017) finds in soils; caring for water acknowledges how humans, fishes and water are interdependent in a wider sense. Correspondingly, as we explore below, if we understand aquariums as life-support systems, we must also recognise that it is not only fishes’ lives that these systems support. To paraphrase Puig de la Bellacasa (see also Krzywoszynska, 2019), the human carers – in this case laboratory technologists and biomedical researchers, but also by extension all those who rely on the knowledge produced through animal research – depend on the water quality’s ‘capacity to ‘take care’ of a number of processes that are vital to more than [ … their] existence’.

In the sections that follow, we take each of these arguments – that (i) humans are capable of attuning to fishes and that (ii) we need a media-specific (here aquatic) approach to such attunements – as a starting-point for thinking through human-fish relations in the laboratory aquarium. First though some more background on research aquariums, their inhabitants and our encounters with them, will help to situate the analysis.

## 3. Zebrafishes in animal laboratories

The animal research laboratory might not be the most obvious setting through which to explore relations between humans and fishes, at least in comparison to work that has examined fisheries (Giraldo Herrera, 2022; Palsson, 1988; Paterson et al., 2014), aquaculture (Law & Lien, 2013) or angling (Bear & Eden, 2011; Browman et al., 2019). Yet it represents a site of rapid and relatively recent change where an ever-greater number of people are brought into contact with an even greater number of fishes. While a variety of fishes (and other aquatic species) are used in laboratory science, by far the greatest vector in this process has been the emergence of zebrafish as a key model organism in the biosciences (Lidster et al., 2017; Teame et al., 2019). In fact, in terms of regulated procedures in the UK and Europe, zebrafish tend to vie with rats for the place of second most used vertebrate species overall (behind mice) and have often been described as the new lab rat. They are particularly favoured as models for developmental biology and neuroscience, as well as being increasingly used in cardiovascular research and pharmaceutical toxicology. Notably, some of the qualities that seemingly ‘alienate’ zebrafish from humans and other mammals are those most prized by researchers: for example, their eggs are fertilised ex vivo and their embryonic stages are translucent (making it easier to study biological development).

**Figure 1.**
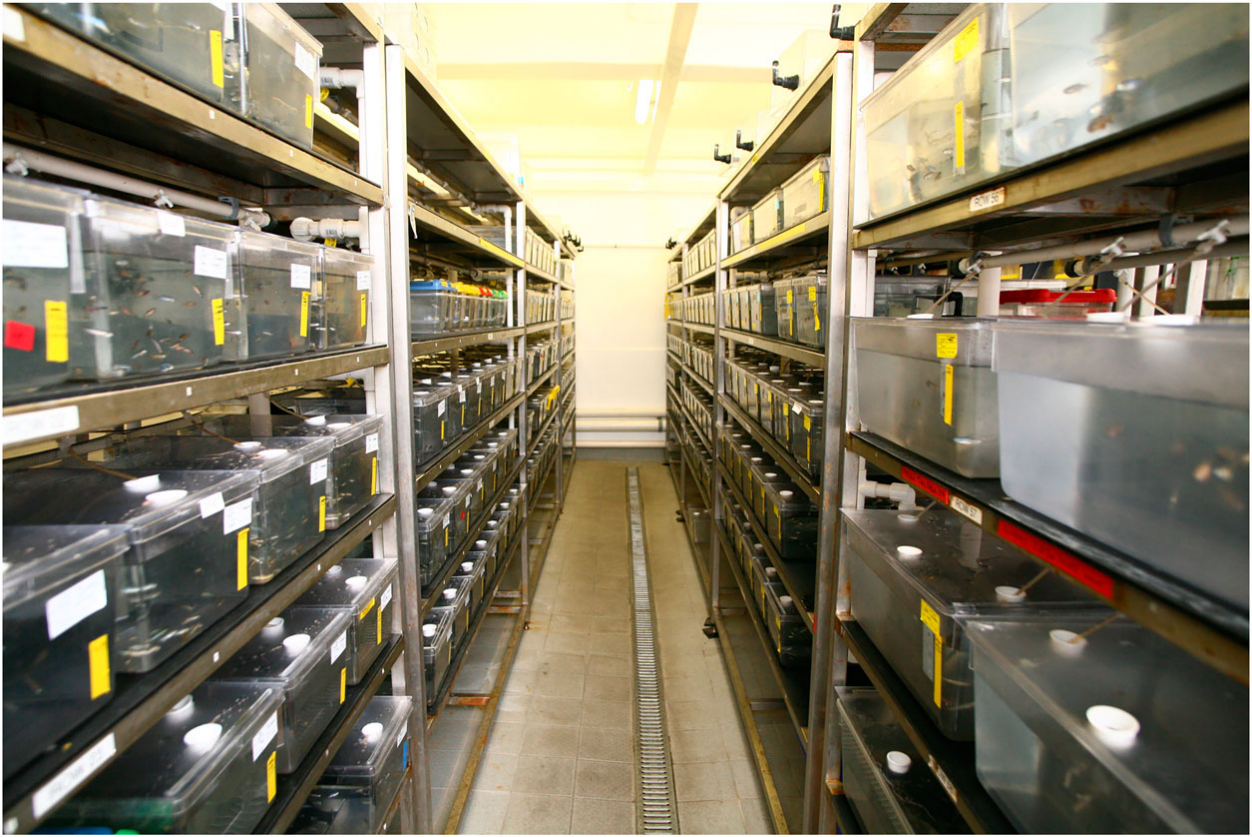
Image of laboratory fish facility with racks of fish tanks. Image from *Understanding Animal Research* (https://www.understandinganimalresearch.org.uk). Reproduced under Creative Commons Attribution 4.0 International license.

As zebrafish have grown in popularity as a model organism, what may once have been a tank or two on a researcher’s desk has burgeoned into large, high-tech aquarium facilities (see Figure 1). A short extract from our research notes gives a sense of what a fish facility looks and feels like:

… aquariums are characterised by serried ranks of plastic tanks on shelves. Tanks regularly contain about 30 fish, which as adults are about half the length of a human’s small finger. It’s warm, as zebrafishes are a tropical species. There’s a constant sound of machines monitoring and adjusting water quality, dosers, pumps, trickling water. Light levels are variable. Tanks have stickers conveying information about genetics and fish health or welfare status. You’ll hear animal technicians and occasionally scientists move around performing various duties, cleaning, feeding, testing, catching, breeding, euthanising … In some, robots feed the fishes. (Composite vignette based on fieldwork notes, 2017-2018)

As our work has traced (Message, 2024; Message & Greenhough, 2019), the rapid growth of laboratory aquariums has entailed significant adjustment, for the researchers working with fish models, the animal technologists who care for them and – we may speculate – for the fishes themselves. Lorimer (2006) suggests that humans tend to be more sympathetic to species that occupy similar ecological niches, that are land-dwelling, diurnal and visible to the human gaze – all of which rarely apply to aquatic species in the wild. The laboratory changes this relation to some extent. In the pursuit of scientific objectivity, zebrafishes are isolated from both their worldly environmental context and other species with which they may co-habit in the wild (compare Fredriksen, 2019), and they are also brought into relation with the interspecies laboratory environment: with human caretakers, live feeds like brine shrimp or rotifers, and occasionally algae-cleaning snails. Racks of clear plastic tanks (see Figure 1) bring the fishes up to ‘our level’, whilst we humans stay with our feet on land, not in water. This design supports close human visual monitoring as zebrafishes become sensible to humans in ways unlikely to happen in other watery sites and spaces such as the shallow rivers of South Asia where zebrafishes originate. This makes these fishes visible and sensible to humans in novel and interesting ways (compare Hayward, 2010). Thus, as Kelly and Lezaun (2017, p. 393) observe, such experimental spaces can provide ‘the scaffolding for a distinct interspecies encounter’ (see also Greenhough, 2012; Haraway, 2008, 2017).

Furthermore, the socio-legal and ethical setting of the animal research facility challenges the assumption that the lives of fish are marginal to and alienated from the lives of humans and other mammals. The rationale for their use as an experimental model is derived from their biological proximity to humans, allowing their physiology to be substituted for that of humans (Svendsen, 2022). Fishes research is governed by the regulations for the use of vertebrates in research. In animal research facilities in the UK, and most of the rest of the world, zebrafish larvae five days post fertilisation are legally ‘animals’, meaning that they, even at three millimetres long, have full legal protections against unnecessary cruelty in the same way as do dogs or rabbits (Message, 2023; Sneddon, 2018). These moments of biological and legal proximity between fishes and humans and other mammals are part of the conditions in which laboratory workers must learn to care for and ‘attune’ to fishes.^5^

Our understanding of these complex and seemingly contradictory legal, ethical, cultural, and practical relations between humans and fishes in animal research laboratories is informed by ten years of ethnographic research in animal research facilities.^6^ Our data includes some preliminary interviews (*n* = 2, including 1 group interview) with animal technologists working in aquatic units undertaken between 2013 and 2015, and a further 21 interviews conducted between 2017 and 2018. These latter interviews involved 27 different individuals, including aquatic facility managers (*n* = 7), animal technologists (responsible for animal husbandry and some procedural work, *n* = 7), researchers working with fishes (*n* = 8), and individuals involved in regulatory and veterinary aspects of fish research (*n* = 5).^7^ Most of our respondents were employed within academic research at universities, but our sample also included some people working in the private sector (pharmaceutical manufacture and regulatory ecotoxicology testing). These interviews were supported by two one-week long periods of participant observation at two large aquarium facilities (conducted by Reuben), as well as numerous other informal visits and participation in professional and industry events over the course of our research. In this analysis, we focus on two themes emerging from this data including: (i) the challenge of attuning to and empathising with fishes and their welfare (Section 4); and (ii) monitoring environmental parameters as a substitute for attuning to fish welfare and behaviour (Section 5).

## 4. Amphibious ethics: attuning to zebrafish in the laboratory

In this section we explore how focusing on the phenomenological experience of relations between humans and fishes, as well as radical physiological differences and evolutionary histories, has culturally shaped and perpetuated fishes’ marginalised social status (Price, 2004). If we follow Helmreich (2009) in arguing that the alien life form is merely one which is not yet familiar in our form of life, how do we develop a more amphibious ethical mode capable of criss-crossing from warm-blooded land-living mammals to cold-blooded aquatic fishes?

It is important to recognise that it is far from easy to incorporate fishes into our human and mammalian form of life without inappropriately erasing their difference or singularity. This issue is intimated by Erica, an experienced aquarist:

They’re so different, the way they behave, the way they move, they don’t look you in the face, so they don’t look at you. They can see you, but they don’t look at you, so yeah, there’s like a bit of a disconnect. (Erica, interview, 2018)

Aquarists emphasised what the human-fishes relationship lacked in comparison to relations with mammalian species, including the fact that you cannot touch and hold fishes in the same way as you would a mouse or rat; fishes are not cute and cuddly, they are not a ‘pet pet’ (Rick, interview, 2015).

Another important factor is the sheer scale of fish facilities, both in terms of the number of tanks and the number of fishes in each tank. It is not uncommon for a fish facility to contain many hundreds of tanks and hundreds of thousands of individual fish, which acts as an emotional and practical impediment to developing bonds with individual fish (Davies, 2012). John (interview, 2013), a facility manager describes how ‘there’s 5,000 tanks’ and ‘a little robot that goes up and down and feeds all the fish six times a day, every day’.^8^ Furthermore, the tank and the water it contains are conceived as a ‘physical barrier’ (Carrie, interview, 2013) to interaction and, for some, to empathy. Some aquarists were attracted to working with fishes precisely because of the perceived ease with which a degree of emotional detachment could be maintained:

I think I find it easier to kind of separate myself from fish because there’s like that layer of glass and you don’t physically interact with them the same way, you can’t pick them up and hold them … so it’s easier to kind of detach yourself a little bit emotionally from that fish. Whereas with mice I think I’d find that a lot more difficult. (Gemma, in a group interview with Fiona and Eugenie, 2018)

Similarly, some aquarists reported that they find it easier, in emotional terms, to kill fish than to kill similar sized rodents. This feeling could be due to the main method used, a massive overdose of anaesthetic in the water, which is perceived to be more humane than some of the methods used to cull rodents such as exposure to carbon-dioxide in rising concentrations^9^ or viscerally less hands-on that physical methods used to cull rodents, which include cervical dislocation, decapitation and concussion. It is also partly because, well, ‘fishes are just fishes’ aren’t they? (see Message & Greenhough, 2019). As Faye notes, we have grown up to think of their deaths as uneventful (compare Roe & Greenhough, 2023): ‘It’s not like when mice are in a gas [chamber …], you see them like suffering; with fishes, it’s like very smooth … and with fishes, most people they grow up like going fishing’ (Faye, interview, 2015).

This experience of disconnection shapes how aquarists relate to fishes, especially given the relatively weak (compared to mouse) scientific evidence base for understanding what a good life might look like for most fish species. While animal welfare regulations assume fishes suffer, sentience in fishes is a relatively new concept in science. Indeed, expert opinion still straddles a spectrum, from those who see fishes as entirely lacking in conscious experience to those who see them as individuals capable of experiencing higher emotions, including pain, boredom and joy (for a state-of-the-art review, see Mason & Lavery, 2022).^10^ Most would agree, however, that, if there is an experience of what it is like to be a fish (to adopt the philosophical idiom), this experience is quite unlike the experience of human, or even mammalian, subjectivity. Reflecting this presumption, aquarists to whom we spoke suggested that, because fishes do not behave like humans or other familiar mammals, their suffering may be invisible to humans:

[F]ish when they are in pain … they don’t express it in a way that humans are initially capable of identifying as pain. … [I]f a person is in pain, they’re either going to make a face or cry or hold whatever bit of them that’s hurting. If your dog’s in pain, it’s going to be acting like oddly, like whining or odd behaviour like not playing with you or something like that or limping, those are obvious signs. Fish don’t do any of that. (Erica, interview, 2018)

**Figure 2.**
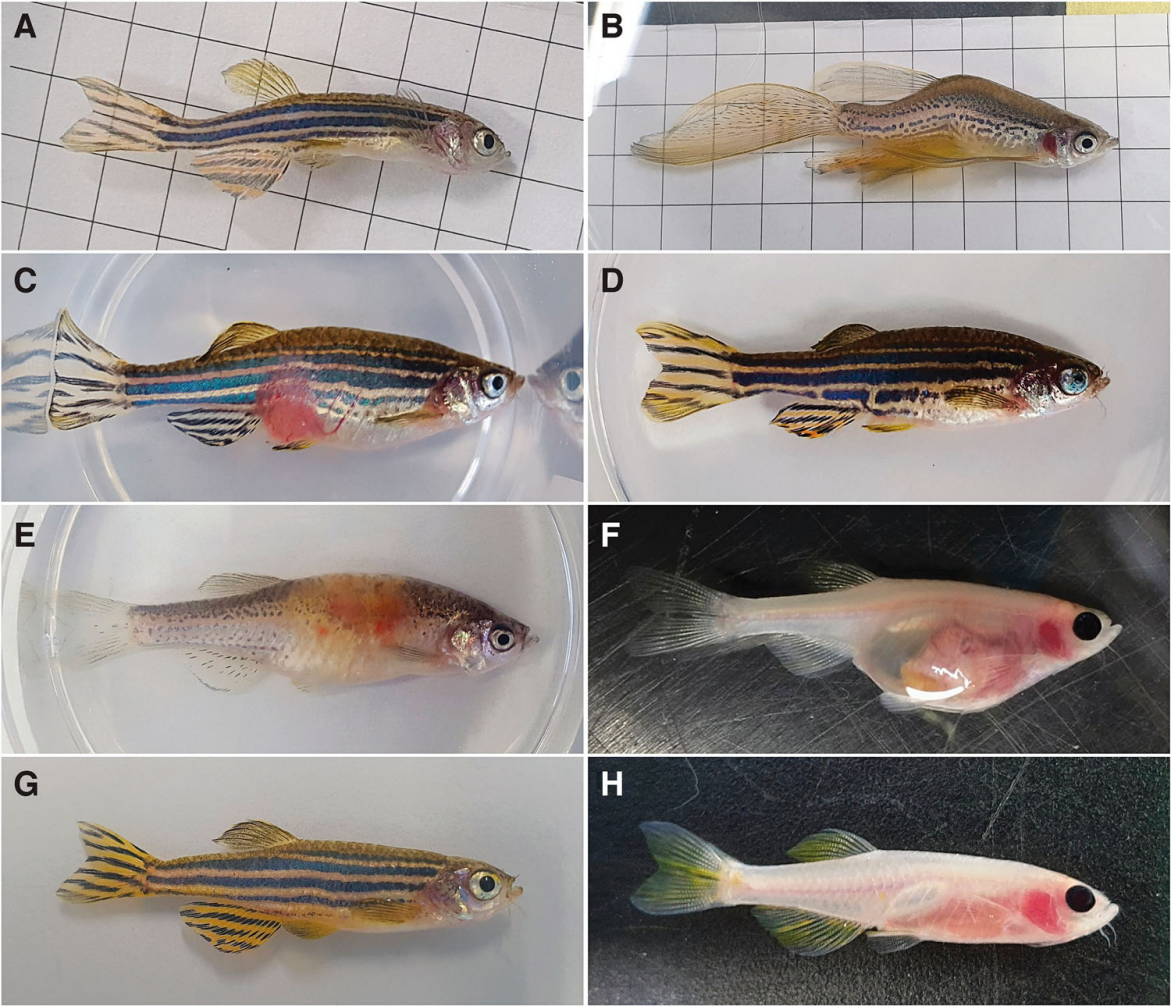
A set of images designed to teach those working with fish how to recognise common illnesses. Images A–F all show fish with signs of illness. Images G and H show two different strains of healthy fish. Source: Sabrautzki et al. (2021). The publisher for this copyrighted material is Mary Ann Liebert, Inc. publishers. Reproduced with permission (reference 5817130093247).

To be marked as different in this way (for instance, lacking an expressive face to relate to) can have several implications for those who may seek attune to and care for the body in question. For humans squinting into fast moving schools of tiny fishes, it takes long-exposure and training to develop the skilled eye necessary to perform the obligatory daily ‘health checks’. Fundamental to this task is determining whether an individual fish is exhibiting behavioural or physical signs consistent with suffering. Manuals and guides support aquarists’ efforts to make judgements about the visual aesthetics of fish bodies and behaviour as consistently as possible: For example, Figure 2 shows a resource developed to teach aquarists how to identify signs of (ill)health in laboratory zebrafishes.

Through experience, and through such technical aids, skilled aquarists learn to ‘read’ fishes’ bodies and to spot signs of health and illness, at least when these are far advanced:

[T]here’s categories, so if we think actually it’s not interacting with any of the other fishes, that’s usually a no-go. If it’s floating, if it’s lost its sense of balance on one side, if it’s down at the bottom, if it’s panting, so if it’s breathing heavily. Obviously, if there’s any massive sores on the body. And then the scales, so sometimes … the scales rise up as well and it’s absolutely horrible to look at … so you’re just like, yeah it’s gone. (Gemma, group interview, 2018)

In the process of applying this skilled assessment, however, we also see in Gemma’s words the beginning of something else, a recognition that witnessing fishes suffering makes her uncomfortable. After all, the desire for detachment described above also implies there is still some capacity for attachment that precedes this. Thus, whilst Chatigny (2019) claims that the more embodied, intuitive forms of empathy that shape, for example, human-dog relations appear inappropriate as a basis for welfare ethics in the case of laboratory fishes, could this instead be symptomatic of the cultural marginalisation and structural de-valuation of fishes?

When supported by the legal and ethical guidelines that underpin all regulated scientific work using fishes, members of staff in the aquariums and laboratories visited for our research were keen to stress their conviction that fishes do indeed suffer in ways at least distantly relatable to us, adopting a kind of strategic anthropomorphism (compare Bennett, 2010; Greenhough & Roe, 2011). Those who worked as aquarists were aware that this conviction set them apart from those who worked with warm-blooded mammalian species or the *‘*fluffies’, as some jokingly called them. As Gideon (interview, 2018), a biomedical researcher with a lot of experience of zebrafish work, argued:

… the more we learn about animals, the more we learn that they’re not that different [to] us. And fishes are the same, and I don’t know why the humans think they’re so special, but fishes have a lot of the same traits as mice or humans.

Similarly, Errol (interview, 2018), an experienced aquarist, asserted that:

I’m of the opinion that a fish is not what used to be looked on as being a wet insect. A fish has feelings, a fish can show you different things. So again, along that line in some ways, a fish is no different from a mouse in how you look at the animal and how you try and – you have to perceive how it is. Again, it has been shown that fish prefer to be in their family groups, they breed better, they coexist much easier. So if they can acknowledge each other, therefore – … there is definitely a form of connection there, that’s what I think.

By articulating these shared points of connection that look beyond human-fish differences, aquarists and socio-legal and material apparatus of the laboratory contribute to a cultural conviction that fishes are living, sentient beings. In other words, following Law and Lien (2017, p. 30), ‘we approach sentience not as a property of fish (or people) as such, but as a relational quality that emerges within heterogeneous assemblages of all kinds’, including those formed within laboratory aquariums. This is a move that we find resonates with other spaces where human societies are learning how to be affected and socially-inclusive towards bodies differing from the white, western, male norm (Ahmed, 2007). This move in turn supports a culture of active inclusivity, one which begins with the convictions that fishes do suffer and that humans have the capacity to recognise and respond to this suffering. Similarly, Hayward (2010; see also Bear, 2011) describes their shifting sensibilities towards cup corals developed through their time spent as a participant observer in a marine research lab, notable in the development of enhanced sensitivity towards the embodied and tactile dimensions of visual perception based on attempts to think/feel like coral, what they term ‘fingeryeyes’. Both corals and Hayward are changed by time spent in the lab together in spite of the acknowledged power dynamics of the laboratory environment, ‘becoming more than ourselves’ (Hayward, 2010, p. 593).

Sensibilities that reach across species differences – that begin with what is held in common, rather than what divides when learning how to care well or better – go beyond the objective and pragmatic process of health assessment. Something beyond a check-box list of ‘things to look for’ motivates ethical concern. As Evelyn (interview, 2018) describes it:

… there is always a nagging doubt in the back of my head, there always will be. My dad was a fisherman, you know, and you can’t tell me that having a hook through your lip is not going to be painful. Can they feel it? I don’t know.

This sense of ethical responsibility towards fishes that suffer came across strongly in Erica’s (interview, 2018) description of the care involved in moving fishes by tank or net:

So basic things like, *‘*How fast do you walk with a tank in your hand?’ Because if you’re walking really fast, it starts sloshing around and that stresses them out and that’s going to be an unpleasant experience for them. There’s nothing regulated, there’s nothing in ASPA^11^ that says you can’t do that, but you know … or you sometimes see people … and they stand there because they’re talking to someone, they’ve got a fish in their net and they’ve forgotten about it and they’re standing there, chatting. It’s like that’s a very bad example of understanding that, that is something alive and what you’re doing is having a negative impact on it.

For some – if not all – this ‘sensibility’ provides an impetus to change things. Uninhibited by gaps in scientific knowledge about what makes fishes ‘happy’ or relieves their ‘boredom’, some of the facilities that we visited provided environmental enrichment, introducing artificial plants, tunnels or houses, live food and scanned images of substrate and aquatic environments attached under or between tanks. We observed how the aquarists subsequently noted changes in behaviour amongst the fishes in their care. Enrichment seemed effective in ‘helping to deal with aggression in the tanks’ (fieldwork notes, August 2015). Such experiences encouraged these aquarists to adopt and share these practices more widely with colleagues working at other sites and institutions. Without discounting the very real welfare gains that these kinds of interventions are likely to offer, they also navigate between the legal status of fishes as deserving of equivalent care to mammals and the dominant cultural representation of fishes as alien, unknowable and insensitive. This navigation may in turn support establishing ethical concern about fishes beyond the laboratory:

… you can go in a pet shop and buy your little fishes that you have there in an aquarium, and they put it for you in a plastic bag and you can just take it by hand and drive home with it in a plastic bag. And nobody will ever question that: whether that is good for the species or bad for the species; whether it’s a tropical one or a moderate temperature one. Nobody cares. (Hanna, biomedical researcher, interview, 2018)

Finally, it is important to note that these examples of inclusive acts of care are also registered on the bodies of technicians. Spending hours each day health-checking fishes is difficult, embodied labour through which humans’ and fishes’ wellbeing becomes entangled. As Jue (2020, p. 27) notes, ‘just because a variety of perceptual worlds may exist specific to individual animals does not mean that there is not overlap and ecological vulnerability’. Fishes’ bodies are subject to harm and death as they act as models or substitutes for humans (Svendsen, 2022), but human bodies are subject to their own challenges in laboratory aquarium environments, which included: a case of severe OCD connected to the pressure and responsibility of caring for thousands of fishes within a technologically complicated system in which small errors could lead to serious losses; the development of skin problems associated with spending long hours under dim lights; and most commonly of all, fatigue and eye strain through the repetitive labour of paying close attention to small swimming bodies whilst looking for signs of pain and distress. In some cases, aquarists may also contract rare zoonotic diseases (Byers & Matthews, 2002). Both humans and fishes are vulnerable – albeit in different ways – to the laboratory environment, offering further potential grounds (or perhaps waters?) for empathy.

## 5. Tank craft: aquarist practice as speculative immersion

Laboratory aquariums are distinctive from the mouse rooms and other animal research spaces in which animal technologists work. Humans exist in a separate environmental media from fishes; fishes occupy it differently; fishes do not breathe the same way that humans do; water enters through their mouths but leaves through their gills where masses of tiny blood vessels extract dissolved oxygen. In the process of setting up their new fish facility, Errol, Frances, Gwen and Harry (interview, 2018) reflected on what makes fish rooms different.

I mean coming into a totally new set of racking facilities and the environment is completely different [to rodent housing], I mean you walk into a room and [feel] for yourself it’s much more humid than you’re used to. There’s a lot of little things but they all add up to making it different … it’s not normal and we just have to adjust ourselves and our parameters to what is normal for the fish.

Laboratory aquariums are both distinctive aquatic milieu (Jue, 2020) or webs of relations (Law & Lien, 2013) and distinctive laboratory spaces where both fishes and the humans working with and caring for them live hydro-social lives (Liao, 2024). Laboratory aquarium design – fishes housed in transparent tanks, placed at human eye level – contrasts with aquatic designs of fish farms, where farmed fish are usually housed in pools on land, or netted pools in lakes or fjords, where fish-farmers either peer over into murky, camouflaging water from gang-planks or use remote underwater cameras. In contrast to the amphibious modes of dwelling that characterise (some of) the inhabitants of Hazard’s (2022) riverscape and Anand’s (2023) anthroposea, humans are prohibited from sharing fishes’ environments in research facilities: they cannot join the fishes in their tanks. In our fieldwork, we heard a lot of statements contrasting experiences of caring for rodents, who breathe the same air and sense the same temperature as humans, and caring for fishes: ‘So if you go into a mouse room and something’s wrong with the air you feel it yourself. Here, if something’s wrong with the water, you can’t*’* (Fae, interview, 2018).

Furthermore, as Errol (interview, 2018) suggests below, fairly minor environmental changes for warm blooded terrestrial mammals – changes in temperature for instance – can be fatal for some fishes:

[With] the [mammalian] animals, if the temperature goes up and down or the humidity goes up and down, it doesn’t have an immediate effect, unless it’s really, really drastic to like minus one. With the fishes, because they are completely literally immersed in their environment, it has a bigger impact on these animals.

Such an approach is consistent with welfare research on fish physiology. For example, Huntingford et al. (2006, p. 333) suggests that:

A number of key [physiological] differences between fishes and birds and mammals have important implications for their welfare. Fishes do not need to fuel a high body temperature, so the effects of food deprivation on welfare are not so marked. For species that live naturally in large shoals, low rather than high densities may be harmful. On the other hand, fishes are in intimate contact with their environment through the huge surface area of their gills, so they are vulnerable to poor water quality and water-borne pollutants.

Indeed, fishes in general are famously and proverbially dependent on their environment: we do not refer to ‘fish out of water’ for nothing!

Consequently, caring for fishes in indoor aquariums demands different modes of engagement, attunement and attention, because they are in an environment ‘outside’ of that the human carers are experiencing. Like the ocean, the aquarium as lived-in space is made accessible to humans via ‘chains of mediation’ (Jue, 2020, p. 3; see also Liao, 2024) and within the aquarium technologies offer readings that serve to render sensible various aquatic parameters – temperature and water quality, salinity and acidity – that humans are unable to sense independently. During his fieldwork, Reuben came to think of aquarists as sometimes like ‘a cross between a plumber and an engineer’ (fieldnotes 2017-2018), sustaining life through servicing life support systems. Some aquarists were attracted specifically by the technical challenges of working with fishes:

… this stuff now is technical stuff and it’s high tech. Everything is computerised, you know. We normally put [in] salt and chemicals and keep the water parameters by computers actually injecting whatever they need to inject, pH or salts or, you know, checking of the water parameters … and oxygen levels and everything. We are only here to control that stuff from the point of view of technology’ (Rick, interview, 2015).

As Fredriksen (2019, p. 772) observes in the context of marine science, the use of technologies and monitoring devices to observe fish in their own worlds is imperfect, smoothed out and abstracted, but it is still affective; a means by which fish may make their presence felt. Furthermore, the kinds of expertise being acquired and discussed by aquarists are not only scientific but pertain also to animal husbandry, analogous to the ‘hobbyist’ knowledge discussed by Muka (2022) in her study of reef tank builders in the aquarium hobbyist community; what we might term, drawing on Muka and paraphrasing Krause (2014), ‘tank craft’. The ‘high tech stuff’ described by Rick acts to sensitise human bodies to fishes’ environments. This is not to say that technical data (water chemistry and change rate, room temperature, lighting levels and cycles, air quality) is not a feature of animal research rooms housing mammalian species, but rather that in the absence of a presumed shared bodily experience of cold/heat/light/smell, the readouts from water quality monitoring equipment take on a very prominent role.^12^

Caring about water quality is not only about caring for fishes but also about caring for good quality science: reproducibility in zebrafish research is becoming a prominent concern and variations in water chemistry between labs are recognised as an important driver of this reproducibility:

… they’re heavily reliant on their environment in a way that mammals aren’t. So you change their environment, you change them internally. And that’s a real problem for –, people can’t get [it] –, it’s hard to understand that really, and of course it has a massive impact on science. (Fae, interview, 2018)

For researchers, this attention to the media-specificities of caring for fishes in aquatic environments also demands adjustments to regulatory requirements, guidelines and experimental protocols. Here’s Hanna (interview, 2018), a biomedical researcher, describing why the aseptic surgery techniques required by Home Office regulators are to date, in her view, not adapted for work with fishes:

And if you start now introducing aseptic injury techniques, you extend the period where the fish is basically handled, and some aspects of it require that you treat the fish with substances that you should not treat fish with. Because they’re harmful to the skin and they, they are actually very nasty [toxic] if you put them in the water.

For some, this focus on environments instead of bodies can be seen as emotionally distancing, making it easier to reconcile the work of caring for fishes with their experiences and eventual fate as laboratory models. As we noted in section four, several animal technologists told us that working with fishes is not like working with mice:

It’s weird, because it’s not –, like you get to interact with the mice because you see them every day. I go in there and I have a conversation with them … But with the zebrafish and the trout, it’s just you can’t do much. You just go in and clean their tanks, and you feed them, you measure the temperature, do the tests and that’s it, that’s all. (Debbie and Fiona, interview, 2013)

However, as we have seen, this does not mean that somatic sensibilities never come into play. Time spent with a specific species and a technological assemblage designed to sensitise humans to the complex interplay of fishy bodies and aquatic environments, allows what Jue (2020, p. 3) terms a milieu-specific analysis to emerge, ‘calling attention to the differences between perceptual environments and how we think within and through them as embodied observers’. For Jue, milieu-specific analysis is a form of situated knowledge (after Haraway, 1988) which ‘acknowledges that specific thought forms emerge in relation to different environments, and that these environments are significant for how we form questions about the world’ (Jue, 2020, p. 3). This form of ‘tank craft’ is also recognised by those who work with fishes in animal research, tying together care for the science noted above with care for fishes and the aquatic environments within which they dwell. For example, one researcher bemoaned the lack of attention paid to water quality in research publications: ‘you know, there’s a lot of–, a lot of studies where there’s no kind of reporting of pH levels or adjustment of pH levels and things’ (Gerrie, interview 2018).

We contend that this close attention to the entanglement of aquatic dwelling and fishes’ lives also serves to attune aquarists towards not only environmental wellbeing. At the outset of this paper, we noted how postcolonial authors like Price (2004) have argued that long-standing ignorance about aquatic life has been strategic to easing how waters – presumed empty and holding no previous claims of ownership – could be colonised. In learning from the experiences of aquarists, work on caring in animal geographies’ margins gathers new insights about where, when and for what we have *not* trained our somatic sensibilities to be sensitive towards. We have thus used our interviews with aquarists to understand the laboratory aquarium as both a specific site through which knowledge of fishes and their environments is produced, but also as an intellectual provocation that challenges our terrestrial understandings of what healthy bodies and healthy environments are like.

Many fish species are exquisitely sensitive to changing conditions, leading them to be heralded as animal sentinels, ones whose welfare becomes an indicator of overall ecosystem health (Gramaglia & Mélard, 2019; Hartman-Davies & Lorimer, in press; Lakoff, 2016; Reif, 2011). Equally striking is the conviction that fishes are immersed in their environment in ways that warm-blooded, terrestrial, air-breathing mammals are not. In the wake of growing recognition of the impact of air-pollution on human health (Kenis & Loopmans, 2022), this is a conviction that needs further scrutiny. The key point is not so much to presume that we can ever really know or feel what fishes feel; rather it is to unsettle and provincialise our terrestrial, mammalian, individualised ways of being and seeing as only *one* way in which the world is experienced. This shift is analogous to how moving to a fish facility can unsettle previous convictions and ways of working in animal research facilities that are – as many of our interviewees suggested – made for mice (Palmer et al., 2021). Like rivers, laboratory aquariums can be seen as key testing grounds not only for new medicines and scientific theories, but also for new ideas of, and relations towards, ecologies (compare Waley, 2000). Can the work on keeping fishes in aquariums healthy, which sensitises aquarists to the intimate interconnections between bodies and environments that sustain or endanger health, perhaps offer land-dwelling critters a new perspective on the ecosystem reframed as life support system? Such forms of speculative immersion, we argue, hold the potential to sensitise humans to the experience of fishes in other anthropogenically transformed sites such as fish farms, as well as to our interdependencies within the environments where we humans dwell.

## 6. Conclusions

In her book on human-animal encounters in the laboratory, *Animal Ethos*, Sharp (2019, p. 226) writes about her decision to focus on mammals because of her intuition that they would provoke the most moral improvisations and challenges. However, in closing she explicitly raises doubt about her own preconceptions on this score and essentially calls for thinking ‘like a zebrafish’. As she puts it:

… my attempts to decipher moral thinking in science are informed by myopic understandings of which creatures matter most in the day-to-day of laboratory life and death. Whereas a key premise of my project has been that mammalian life offers a potent entry point for ethnographic engagement, an expanded horizon – one that encompasses other lab-based species that might range from fishes to frogs to slime mould – reveals yet another black box of experimental sentiment, welfare and care.

This paper constitutes one attempt to open up the ‘black box’ of fishes to examine how care takes place where the limits of attunement, corporeal compassion and somatic sensibility are culturally delineated, applicable in our case to both understandings of animal research facilities and fishes. As Giraud (2019) would remind us, with some notable exceptions in the fields of applied ethology and animal welfare science, (Sloman et al., 2019; see, for example, Sneddon, 2011; Sneddon et al., 2014), most experimental protocols conducted on fishes are not produced with the aim of facilitating a better understanding of fishes, how they experience the world and how we should relate to them. Licenses for experimental work with fishes are dominated by forms of work that use fishes as biomedical models or substitutes for human subjects (or components of human subjects), or to inform practices such as aquaculture that seek to exploit fishes for human consumption (compare Liao, 2024). The lives of fishes in tanks, we might reasonably speculate, are far more constrained than those of fishes in the wild, with less opportunities to flourish and thrive even before we take into account the genetic modifications, habituations and other factors that may render them more vulnerable. Indeed, there remains, as Davies (2014, p. 2608) notes, ‘a central absence about the proper way of knowing and living with these new companion species’. However, this statement does not mean that such spaces cannot also be sites for the emergence and development of other modes-of relating (see also Lien, 2015). As Fredriksen (2019, p. 776) notes, even though contact zones are sites where ‘bodies become irresponsible towards one another, spaces not only of unequal power but also of grave bodily and cultural violence’, there remains the ‘possibility of creating a space where the presence of others may make itself known, even if imperfectly. The possibility of responsibility to others may be opened up in encounter’.

Taking inspiration from those who approach relations between humans and fishes with respect, care and responsibility (Giraldo Herrera, 2022; Krause, 2014; Paterson et al., 2014; Todd, 2016), in this paper we have drawn on our conversations with aquarists about how they learn to care for fishes to make two key observations about the possibility of milieu-specific (Jue, 2020) somatic sensibilities. Firstly, zebrafishes offer a good case to address Bear and Eden’s (2011, p. 336) suggestion that we should be ‘more attentive to the heterogeneity of categories of human and nonhuman’ and ‘critical of assumptions that some animals, such as fish, are alien to humans’. A culture that describes zebrafishes’ features, senses, responses and environments as alien presumes little basis for ‘sharing suffering’; and, relatedly, their limited visibility – in real and regulatory terms, as well as in public imaginations – exemplifies their marginal status within hierarchical species knowledge systems. Yet our data suggests (on the part of humans) evidence of practising both alienation and empathy to zebrafishes: expressed indifference and caring conscientiously through difference. While zebrafishes may constitute a ‘limit case’ for somatic sensibilities, the specific material, affective and ethical spaces of the laboratory contribute towards developing a human capacity for caring for fishes that directly employs skilled somatic attunement to the condition of individual fish bodies. Time spent with a species, and the proximity and visibility generated through ‘tank craft’, offers one route towards the development of more nuanced and care-orientated dispositions towards fishes. We suggest that this could form the basis for an ‘amphibious ethics’, which might in turn lead to greater convictions that: (i) cold-blooded species can and do feel pain; (ii) care – in the form of environmental enrichment, for example – matters for fishes; and (iii) partially shared vulnerability within (in this case) the laboratory environment registers across the bodies of humans and animal alike when somatic sensibilities are afforded significance.

The ‘tankcraft’ of laboratory aquarists, like the watercraft of fisherpeople (Krause, 2014), offers promising lines of enquiry for geographers seeking to develop more inclusive concepts and projects in animal geographies that make space for ‘awkward creatures’, culturally marginalised by virtue of their bodies and habitats being so radically other to those of creatures culturally privileged by humans. The potential arises here for allowing us to see laboratory aquariums not as sites where species are set at a distance or ‘out of place’ (Krause, 2014; Wilson, 2019), but rather as sites where things are brought into different kinds of proximity and allowing multiple different kinds of relations to emerge. This is not to say that in order to know fishes and other critters better, animal geographers need to build aquariums or become reef tank hobbyists. Rather, we advocate an approach akin to what Koch and Svendsen (2015, p. 371; see also Message, 2024) describe as ‘moral pathfinding’, a method of research that seeks out multi-species contact zones and ‘traces and highlights how actors grope their way through a changing moral landscape, which is shaped and reshaped concomitantly with their practices and experiences’ (compare Bear, 2019).

Secondly, we also find a strong focus on care for environment, the watery milieu of the fishes, that contrasts with the case of mammals in animal research facilities. Whilst animal wellbeing and environmental harm/care are often set apart or treated separately through a focus on welfare and conservation respectively (for an overview, see Sebo, 2022), the case of laboratory aquariums evidences the inseparability of fishes and the watery milieu which they inhabit and which inhabits them, raising the possibility that somatic sensibilities might extend to remembering the all-round qualities of an environment that sustains good health and welfare, whether air or water or an amphibious mixture. As human relations with the environment, including the oceans, are becoming increasingly intense in terms of exploitation and research, so blue spaces are becoming increasingly ‘lab like’ (Brown & Peters, 2019; Palsson, 1988), sensed, monitored, explored and subjected to ongoing experimental interventions (Alaimo, 2019). Simultaneously, humans’ and fishes’ lives become more and more entangled through practices of exploitation, farming (aquaculture), angling and research. Furthermore, as Neimanis (2012, p. 92) notes, with fishes having a heightened susceptibility to environmental change, ‘[w]hile species extinctions are occurring at around 10% per decade, aquatic species face a higher threat of extinction than birds or mammals’.

In this context, thinking with care in the zebrafish research aquarium offers what Jue (2020) might term a milieu-specific analysis of emergent and changing relations between people and fishes; indeed, a kind of speculative immersion. Significantly, within such analyses the qualities of the animals’ environment become the focus of analytical attention and concern, not just the context through which specific ways of bodily knowing are situated or emplaced. As aquarists and researchers become more sensitised to fishes within the laboratory environment, their relationships also serve as a model for how somatic sensibilities can be extended through both technical sensing assemblages and aquatic media, amending the ignorance linked to the short-sighted, disabling terrestrial bias found widely in human societies (Bear & Eden, 2011; Jones, 2000; Jue, 2020). We draw attention to laboratory aquariums as infrastructures of life-support to make a case for a more amphibious, inclusive approach to more-than-human ethics and animals’ geographies, one that can grow more attentive to and learn from shared experiences, exposures and vulnerabilities in marginal aqueous contact zones.

## Supplementary Material

Rightslink® by Copyright Clearance Center.pdf

## Data Availability

Where permission has been granted by the interviewees, the dataset on which this article draws has been anonymised and deposited in the UK Data Archive, subject to an embargo (Davies et al., 2033).

## Notes

1 Reuben is now a Research Fellow in Responsible Research and Innovation in Science, Technology and Innovation Studies, School of Social and Political Science, University of Edinburgh, Edinburgh, UK

2 It is important to recognise that other knowledge traditions may have diverse and different modes of human-fish relation, maybe grounded in ideas such as care and mutual respect (see, for example, Krause, 2014; Todd, 2016).

3 We use the term ‘fishes’ rather than the more common collective noun of ‘fish’ here as a gesture towards recognising the extreme diversity of lifeworlds and likely welfare needs amongst this enormous taxa, as well as to acknowledge that many fish species, although they may shoal in vast numbers, are likely to have the capacity to experience their worlds as individuals (see also Balcombe, 2017).

4 ‘Murine’ means ‘Old World’ rodents, chiefly mice and rats.

5 Although it is important to note that many in the field feel that formal regulatory alignment does not translate easily into the day-to-practice of caring for fishes. People who work with fishes often feel that their work is marginal to the wider organisation of animal research and its historical and ongoing focus on murine models (Message & Greenhough, 2019). While this situation is changing, some people who work with fishes still feel as though the Home Office (which regulates animal research in the UK) is essentially a society for the protection of mice, with everything else an afterthought.

6 The projects from which this data is derived were reviewed by the University of Oxford’s Central University Research Ethics Committee, CUREC, approval number SOGE 18A-7.

7 Note: We use the term ‘aquarist’ loosely to designate all of those who work with fishes on a regular basis. More correctly used, it designates only animal technologists – not vets or scientists.

8 See Bear (2019) for a similar argument in the context of insect farming.

9 It is important to note that we are not aware of any evidence that death by anesthetic is more humane death than by C0^2^, but there has been a push back against using C0^2^ because evidence suggests that it is inhumane.

10 The controversy about fish pain is longstanding. A core issue has been lack of agreement on what alternate neural mechanisms may support the experience of pain in fishes in the absence of a neocortex, the part of the brain known to be essential in human’s pain perception (Michel, 2019). At the same time, there are good reasons for believing that pain is an evolved mechanism conserved across vertebrates (Sneddon, 2018), and fishes do fulfil key behavioral and physiological criteria that some have proposed for assessing animal pain (Sneddon et al., 2014).

11 The Animals in Scientific Procedures Act (1986) is the main piece of UK legislation that regulates the use of animals for experimental or other scientific purposes.

12 All fishes, of course, are not the same; they are not all equally sensitive to their environmental parameters: ‘We talk about fishes but of course there’s 30,000 species of fishes and not every fishes responds the same. Cold blooded fishes, warm blooded fishes, fishes that are happy to be in tanks, fishes that aren’t’ (Farol, Interview, 2018). One senior aquarist who we interviewed bemoaned the insensitivity of the researchers who kept their fishes in environmental conditions ill-suited to that specific species (a non-tropical species in this case): ‘If you’re looking at the stress response of X species under whatever conditions, your animals are already stressed because they’re living in water that’s too warm and poor water quality. I’ve gone, ‘Oh, look,’ [laughs], ‘this is not good science at all’ (Nick, Interview, 2013).
