# Supplementary material for: Amphibious ethics and speculative immersions: laboratory aquariums as a site for developing a more inclusive animal geography
Source: Scott Geogr J. 2024 Jul 16;140(3-4):449–73. doi: 10.1080/14702541.2024.2378308 (PMC11495481; doi:10.1080/14702541.2024.2378308)
Supplement: Rightslink® by Copyright Clearance Center.pdf [file RSGJ_A_2378308_SM4108.pdf]

CCC | RightsLink

BG

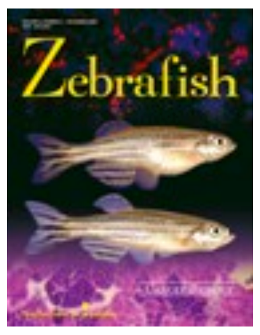

Welfare Assessment of Adult Laboratory Zebrafish: A Practical Guide

Author: Sibylle Sabrautzki, Manuel Miller, Erika Kague, et al
Publication: Zebrafish
Publisher: Mary Ann Liebert, Inc.
Date: Aug 1, 2021

Copyright © 2021, Mary Ann Liebert, Inc.

Order Completed

Thank you for your order.

This Agreement between Beth Greenhough, University of Oxford ("You") and Mary Ann Liebert, Inc. ("Mary Ann Liebert, Inc.") consists of your order details and the terms and conditions provided by Mary Ann Liebert, Inc. and Copyright Clearance Center.

**License number**	Reference confirmation email for license number
**License date**	Jun, 27 2024

Licensed Content

**Licensed Content Publisher**	Mary Ann Liebert, Inc.
**Licensed Content Publication**	Zebrafish
**Licensed Content Title**	Welfare Assessment of Adult Laboratory Zebrafish: A Practical Guide
**Licensed Content Author**	Sibylle Sabrautzki, Manuel Miller, Erika Kague, et al
**Licensed Content Date**	Aug 1, 2021
**Licensed Content Volume**	18
**Licensed Content Issue**	4

Order Details

**Type of Use**	Journal/Magazine
**Requestor type**	academic
**Format**	print and electronic
**Portion**	figures/tables
**Number of figures/tables**	1
**Translating...**	no
**Distribution quantity**	1300

About Your Work

**Title of new article**	Amphibious ethics and speculative immersions: Laboratory aquariums as a site for developing a more inclusive animal geography
**Lead author**	Beth Greenhough
**Title of targeted journal**	Scottish Geographical Journal
**Publisher**	Taylor and Francis
**Expected publication date**	Aug 2024

Additional Data

**Portions**	Figure 2: Examples of fish with clinical signs of illness/abnormalities in comparison with individual healthy fish
**The Requesting Person / Organization to Appear on the License**	Beth Greenhough, University of Oxford

Requestor Location

**Requestor Location**	Keble College, Oxford<br>Keble College<br><br>Oxford, OX1 3PG<br>United Kingdom<br>Attn: Keble College, Oxford

Tax Details

Billing Information

**Billing Type**	Credit Card
**Credit card info**	Visa ending in 5745
**Credit card expiration**	08/2025

Total: 136.11 GBP

CLOSE WINDOW

© 2024 Copyright - All Rights Reserved | Copyright Clearance Center, Inc. | Privacy statement | Data Security and Privacy | For California Residents | Terms and ConditionsComments? We would like to hear from you. E-mail us at customercare@copyright.com

Privacy - Terms
